# Cathepsin K knockout protects against cardiac dysfunction in diabetic mice

**DOI:** 10.1038/s41598-017-09037-z

**Published:** 2017-08-18

**Authors:** Rui Guo, Yinan Hua, Olivia Rogers, Travis E. Brown, Jun Ren, Sreejayan Nair

**Affiliations:** School of Pharmacy, College of Health Sciences and the Center for Cardiovascular Research and Alternative Medicine, University of Wyoming, WY, 82071 USA

## Abstract

Diabetes is a major risk factor for cardiovascular disease and the lysosomal cysteine protease cathepsin K plays a critical role in cardiac pathophysiology. To expand upon our previous findings, we tested the hypothesis that, knockout of cathepsin K protects against diabetes-associated cardiac anomalies. Wild-type and cathepsin K knockout mice were rendered diabetic by streptozotocin (STZ) injections. Body weight, organ mass, fasting blood glucose, energy expenditure, cardiac geometry and function, cardiac histomorphology, glutathione levels and protein levels of cathepsin K and those associated with Ca^2+^ handling, calcineurin/NFAT signaling, insulin signaling, cardiac apoptosis and fibrosis were determined. STZ-induced diabetic mice exhibited distinct cardiac dysfunction, dampened intracellular calcium handling, alterations in cardiac morphology, and elevated cardiomyocyte apoptosis, which were mitigated in the cathepsin K knockout mice. Additionally, cathepsin K knockout mice attenuated cardiac oxidative stress and calcineurin/NFAT signaling in diabetic mice. In cultured H9c2 myoblasts, pharmacological inhibition of cathepsin K, or treatment with calcineurin inhibitor rescued cells from high-glucose triggered oxidative stress and apoptosis. Therefore, cathepsin K may represent a potential target in treating diabetes-associated cardiac dysfunction.

## Introduction

Diabetes mellitus is an independent risk factor for heart failure and is and is characterized by dilated ventricles, hypertrophic cardiomyocytes, pronounced interstitial fibrosis, diastolic dysfunction, and impaired/preserved systolic function, which ultimately leads to heart failure^1–5^. Despite the availability of new drugs to control diabetes, the prevalence of diabetic cardiomyopathy continues to rise. Thus, novel therapeutics directed at the etiology and pathophysiologies underlying diabetic cardiomyopathy are needed.

Recent evidence suggests cathepsin K plays a significant role in the progression of cardiovascular diseases, as well as in the modulation of adiposity and glucose intolerance^6^. Cathepsin K is the most potent mammalian cysteine protease with strong elastase and collagenase properties. Physiologically cathepsin K has been shown to mediate cellular protein turnover, collagen degradation, and the remodeling of the extracellular matrix^7^. In addition, increased expression and activity of cathepsin K has been reported in the hypertrophic and failing heart^8^. We have previously shown that knocking out cathepsin K in mice alleviates obesity and pressure overload–associated cardiac dysfunction in mice^9, 10^. However, the explicit role of cathepsin K in diabetic cardiovascular complications or the potential mechanisms remains unknown. Accordingly, in this study, we hypothesized that cathepsin K knockout protects agains cardiac structural and functional alterations induced by diabetes. We also assessed the effect of deletion of cathepsin K on cardiomyocyte Ca^2+^ handling, oxidative stress, apoptosis and calcineurin/NFATs (nuclear factor of activated T-cells) signaling.

## Results

### Biometric parameters and oxidative stress

As shown in Table 1, STZ-treated WT mice had a reduction in body weight and white adipose tissue mass, without significantly altering the mass of other organs. In contrast, these changes were not evident in the cathepsin K knockout mice. Although liver and kidney mass were unchanged following STZ-treatment, when normalized to body weight, the mass of these organs were significantly increased compared to vehicle treatment, which was attenuated in cathepsin K knockout mice. No significant changes were observed in the heart mass with either the knockout or STZ-treatment. As anticipated, fasting blood glucose levels were elevated in STZ-treated WT mice compared to that of the vehicle control, which was markedly attenuated by cathepsin K knockout. The ratio of reduced-to-oxidized glutathione (GSH/GSSG), a marker of oxidative stress, was diminished in the cardiac tissues following STZ-treatment, which was rescued in the cathepsin K knockout mice.

**Table 1:** General features of C57 and *Ctsk* ^−/−^ mice following 4-weeks of STZ-treatment.

**Parameter**	**WT**	**WT-STZ**	***Ctsk*** ^***−/−***^	***Ctsk*** ^***−/−***^ **–STZ**
Body weight (g)	27.1 ± 0.8	22.7 ± 1.4^*^	25.9 ± 0.9	24.7 ± 0.8
Body weight gain (g)	1.67 ± 0.46	−2.44 ± 0.62^*^	1.29 ± 0.26	−0.66 ± 0.44^*†^
White adipose tissue (g)	1.37 ± 0.09	014 ± 0.06^*^	0.36 ± 0.03	−0.20 ± 0.04
Heart weight (mg)	131 ± 5	117 ± 8	128 ± 9	125 ± 5
Liver weight (g)	1.39 ± 0.07	1.43 ± 0.08	1.37 ± 0.09	1.53 ± 0.06
Kidney weight (g)	0.32 ± 0.02	0.33 ± 0.02	0.36 ± 0.03	0.38 ± 0.01
Heart/body weight (mg/g)	4.73 ± 0.11	5.21 ± 0.14	4.79 ± 0.21	4.88 ± 0.15
Liver/body weight (mg/g)	50.4 ± 1.3	64.1 ± 1.4^*^	51.4 ± 1.6	60.1 ± 2.7^*^
Kidney/body weight (mg/g)	11.5 ± 0.5	14.9 ± 0.8^*^	13.4 ± 0.5	15.0 ± 0.5^*^
Fasting blood glucose (mg/dL)	107 ± 8.5	415 ± 55^*^	110 ± 7.4	287 ± 32^*†^
Water consumption (mL)	5.4 ± 0.25	16.2 ± 2.5^*^	5.6 ± 0.97	19.7 ± 2.6^*^
Cardiac GSH (nmol/mg tissue)	26.9 ± 1.7	24.7 ± 1.1	26.6 ± 1.8	29.9 ± 1.4
Cardiac GSSG (nmol/mg tissue)	14.3 ± 0.9	19.6 ± 0.7^*^	13.3 ± 1.1	17.2 ± 0.8
Cardiac GSH/GSSG ratio	1.95 ± 0.16	1.28 ± 0.07^*^	2.08 ± 0.11	1.80 ± 0.15^†^

Mean ± SEM, n = 6 to 8 mice per group. ^*^p < 0.05 versus WT group; ^†^p < 0.05 versus WT-STZ group.

### Metabolic properties

**Figure 1:**
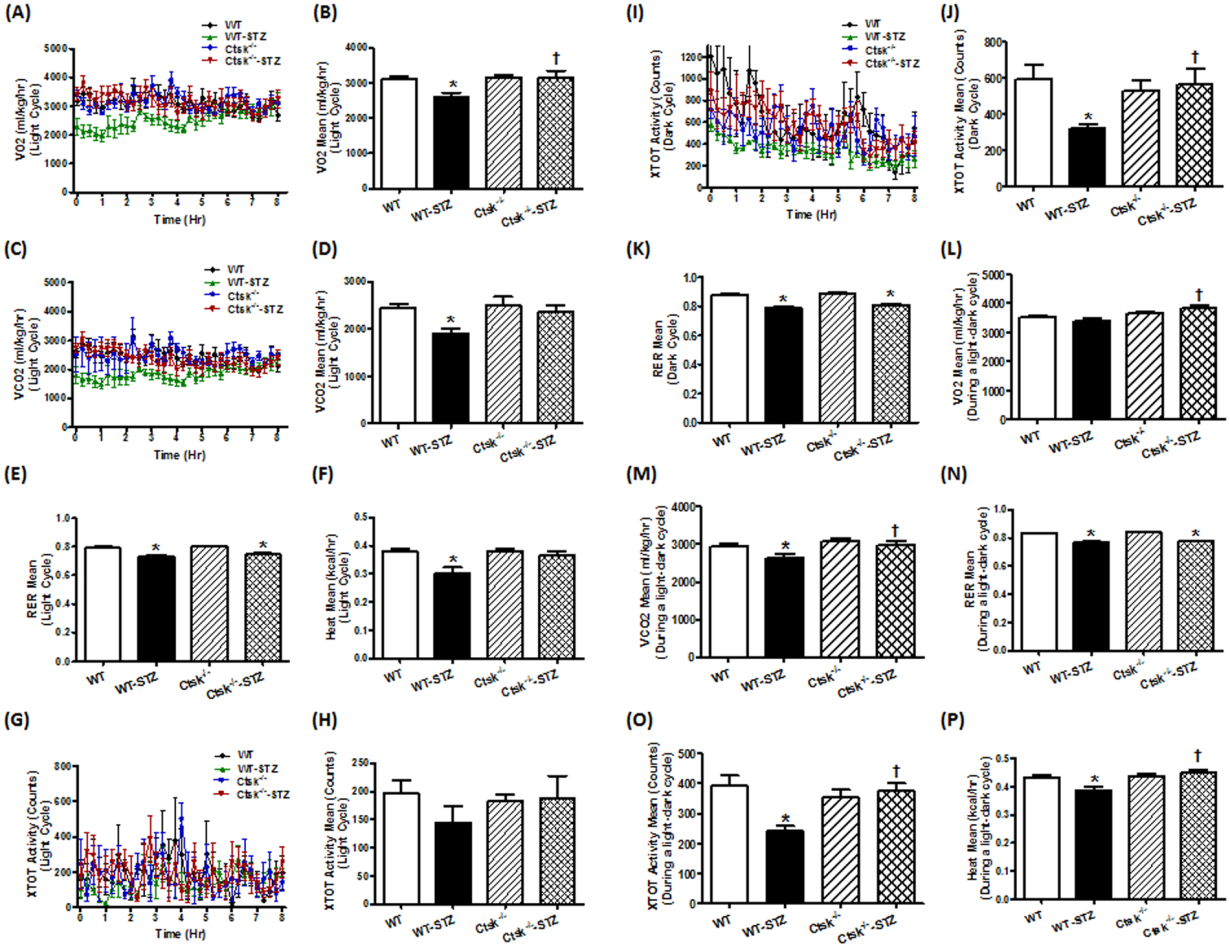
Metabolic properties of wildtype (WT) and cathepsin K knockout (*Ctsk* ^−/−^) mice challenged with vehicle or streptozotocin (STZ) during light and dark cycles. (**A**–**B**) volume of O_2_ consumption (VO_2_) in the light cycle; (**C**–**D**) volume of CO_2_ production (VCO_2_) in the light cycle; (**E**) respiratory exchange ratio (RER) in the light cycle; (**F**) energy expenditure (heat) in the light cycle; (**G**–**H**) physical activity in the light cycle; (**I**,**J**) physical activity in the dark cycle; (**K**) RER in the dark cycle; (**L**) VO_2_ during an entire light-dark cycle; (**M**) VCO_2_ during an entire light-dark cycle; (**N**) RER during an entire light-dark cycle; (**O**) physical activity during an entire light-dark cycle; (**P**) heat during an entire light-dark cycle. Mean ± SEM, n = 5–7 mice per group, ^*^p < 0.05 vs. WT group, ^†^p < 0.05 vs. WT-STZ group

Diabetic mice exhibited metabolic anomalies as evidenced by reductions in oxygen consumption (VO_2_), carbon dioxide production (VCO_2_), and respiratory exchange ratio (RER = VCO_2_/VO_2_), under both light and light-dark cycles. Whereas cathepsin K knockout mice reconciled STZ-induced attenuation of VO_2_ and VCO_2_, which it did not alter RER (Fig. 1A–E,K–N). Furthermore, the total activity and energy expenditure (heat production) were markedly reduced in the dark cycle in WT diabetic mice, which were reversed in the cathepsin K knockout mice (Fig. 1F, I–J, O-P).

### Echocardiographic properties

**Figure 2:**
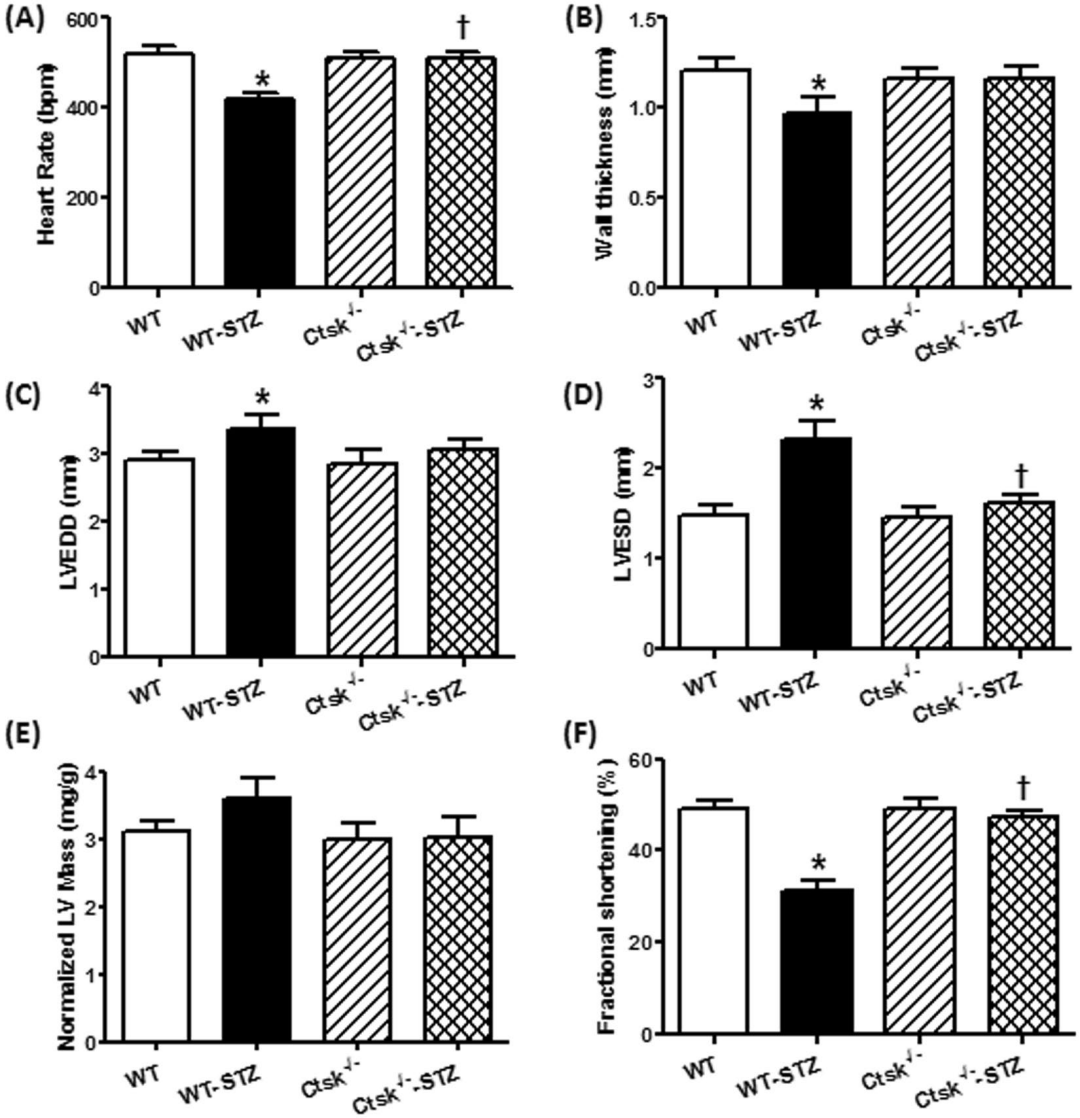
Echocardiographic properties in WT and *Ctsk* ^−/−^ mice treated with or without streptozotocin. (**A**) heart rate; (**B**) wall thickness; (**C**) left ventricular (LV) end-diastolic diameter; (**D**) LV end-systolic diameter; (**E**) LV mass normalized to body weight; (**F**) fractional shortening. Mean ± SEM, n = 6–8 mice per group. ^*^p < 0.05 vs. WT group, ^†^p < 0.05 vs. WT-STZ group.

STZ-induced diabetes caused reductions in the resting heart rate and wall thickness (Fig. 2A–B), as well as increased end-diastolic dimension (EDD) and end-systolic dimension (ESD) (Fig. 2C–D). These anomalies were not observed in the cathepsin K knockout mice subjected to STZ-treatment. Furthermore, STZ-treatment significantly depressed fractional shortening in WT mice, which was reconciled in the cathepsin K knockout mice (Fig. 2F). Both absolute (not shown) and the normalized LV-mass (calculated using echocardiography) were comparable among all groups. However, consistent with its effects on heart mass (normalized to body weight), diabetic WT mice showed a tendency towards increased normalized LV-mass (not seen in cathepsin K knockout mice), which is attributable to a decrease in the body weight in the WT mice following STZ injections (Fig. 2E).

### Cardiomyocyte contractile function and intracellular Ca^2+^ properties

**Figure 3:**
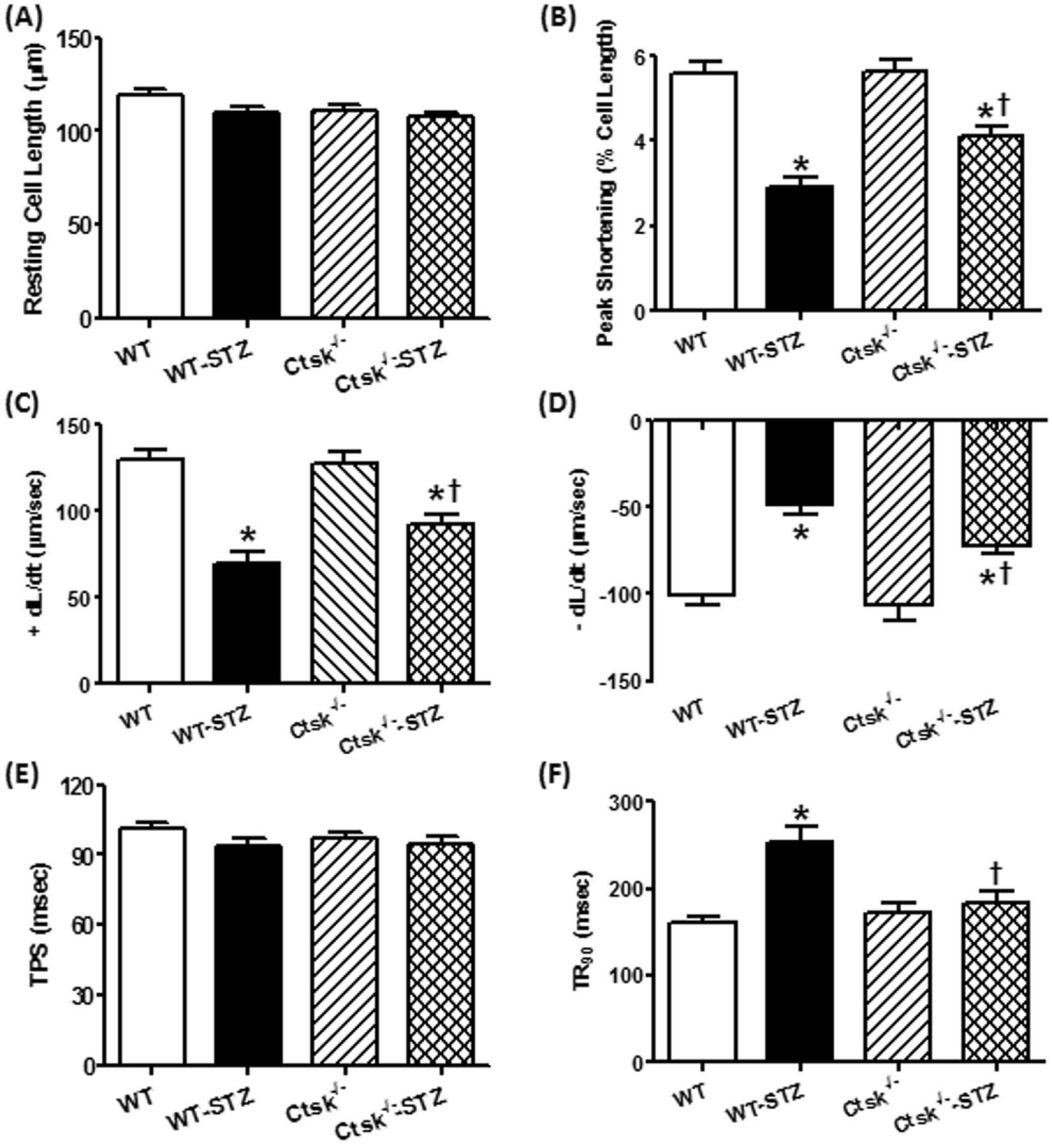
Cardiomyocyte contractile properties in WT and *Ctsk* ^−/−^ mice treated with or without streptozotocin. (**A**) Resting cell length; (**B**) peak shortening (PS), normalized to cell length; (**C**) maximal velocity of shortening (+dL/dt); (**D**) maximal velocity of relengthening (−dL/dt); (**E**) time-to PS (TPS); (**F**) time-to-90% relengthening (TR90). Mean ±SEM, n = 92–97 cells from three mice per group. ^*^p < 0.05 vs. WT group, ^†^p < 0.05 vs. WT-STZ group.

**Figure 4:**
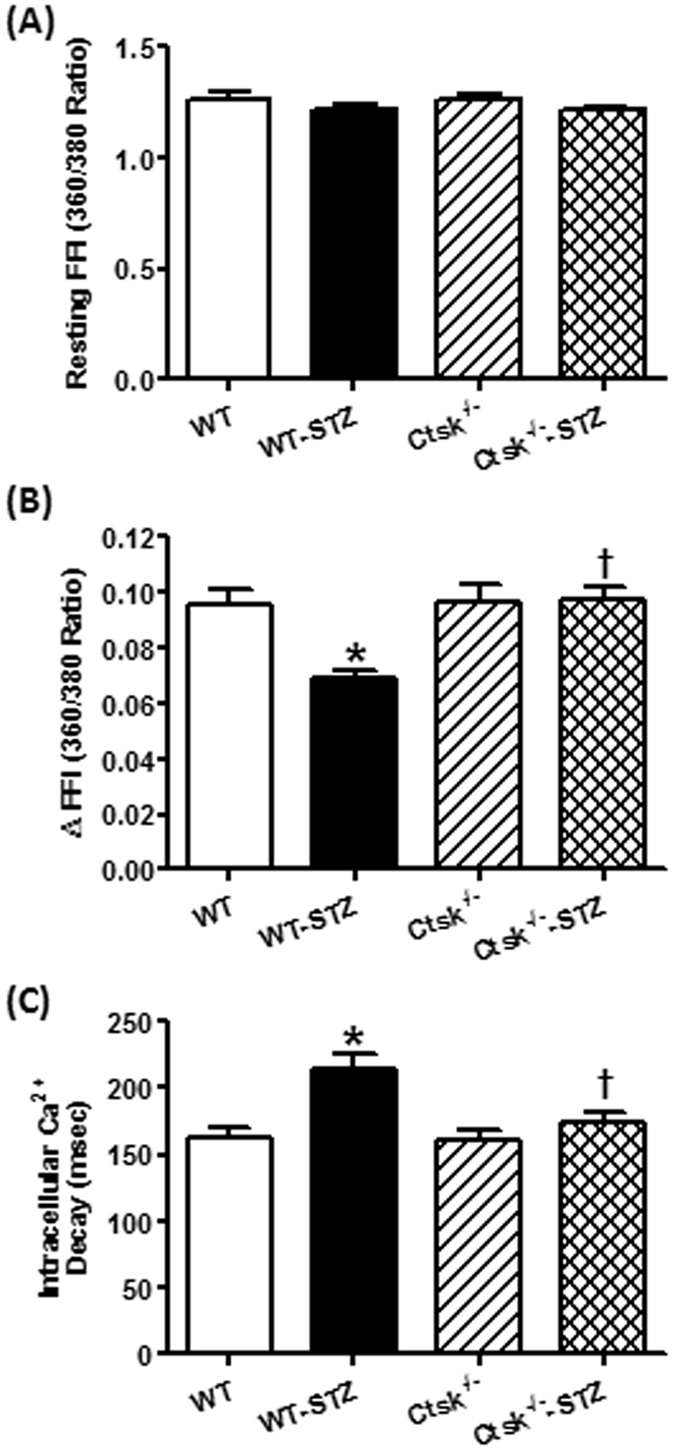
Cardiomyocyte intracellular Ca^2+^ handling properties in WT and *Ctsk* ^−/−^ mice treated with or without streptozotocin. (**A**) Resting fura-2 fluorescence intensity (FFI); (**B**) electrically-stimulated rise in FFI (ΔFFI); (**C**) intracellular Ca^2+^ decay rate (single exponential); Mean ± SEM, n = 105–120 cells from four mice per group. ^*^p < 0.05 vs. WT group, ^†^p < 0.05 vs. WT-STZ group.

There was no significant difference between STZ-treated and cathepsin K knockout mice with respect to resting cell length or time-to-peak shortening (TPS) in isolated cardiomyocytes (Fig. 3A,E). However, cardiomyocytes isolated from STZ-induced diabetic mice had significantly reduced peak shortening (PS), maximal velocity of shortening/relengthening ( ± dL/dt), and prolonged time-to-90% relengthening (TR90), all of which were markedly diminished in cardiomyocytes isolated from cathepsin K knockout mice treated with STZ (Fig. 3B–D,F). In addition, in cardiomyocytes isolated from diabetic mice there was a significantly depression in intracellular Ca^2+^ rise in response to electrical stimulus (ΔFFI) (Fig. 4B), and reduced intracellular Ca^2+^ decay rate (single exponential curve fit) (Fig. 4C), and an unchanged resting intracellular Ca^2+^ concentration (Fig. 4A). In contrast, cathepsin K knockout negated STZ-induced prolongation in intracellular Ca^2+^ decay and depression in ΔFFI without affecting the baseline FFI.

### Evaluation of cardiomyocyte hypertrophy and fibrosis

To evaluate the impact of genetically knocking out cathepsin K on myocardial histology in diabetic mice, cardiomyocyte cross-sectional area and cardiac fibrosis were examined (Supplementary Fig. 1). FITC-conjugated wheat-germ staining sections revealed increased cardiomyocyte cross-sectional area following STZ-treatment, which indicates cardiomyocyte hypertrophy. In contrast, cardiomyocytes from cathepsin K knockout mice subjected to STZ-treatment resisted hypertrophy (Supplementary Fig. 1E–H,J). Masson trichrome staining revealed the presence of perivascular fibrosis in the diabetic heart, which appeared to be attenuated in cathepsin K knockout mice (Supplementary Fig. 1A–D). Additionally, Supplementary Fig. 1I shows a trend towards attenuation of diabetes-induced elevation in collagen type I content in the cathepsin K knockout mice.

### Calcineurin/NFAT signalling

**Figure 5:**
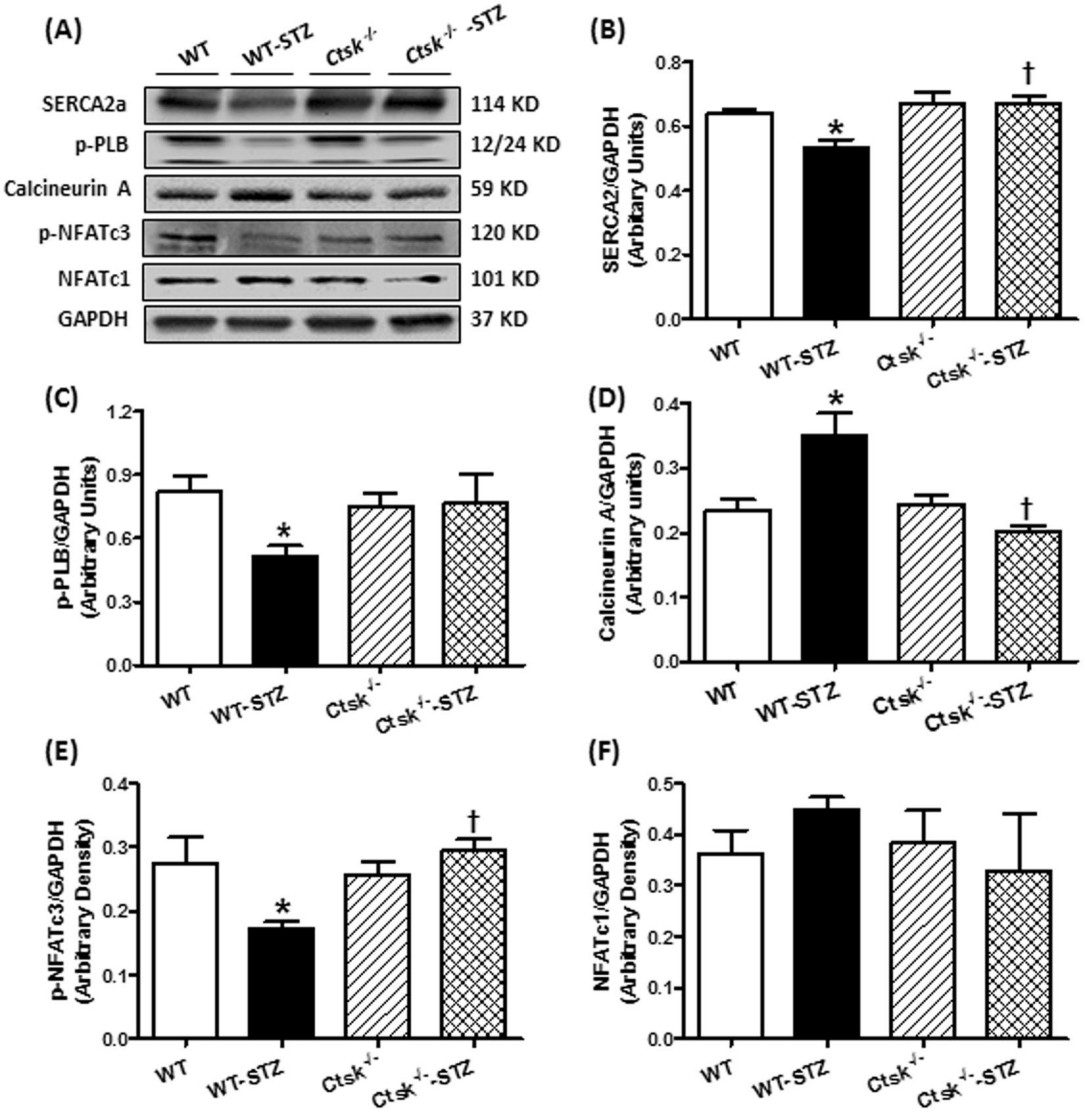
Western blot analysis exhibiting levels of Ca^2+^ regulatory proteins as well as calcineurin A-NFAT signaling in myocardium from WT and *Ctsk* ^−/−^ mice treated with or without streptozotocin. (**A**) Representative gel blots of SERCA2a, phosphorylation of phospholamban (p-PLB), calcineurin A, phosphorylation of NFATc3, NFATc1 and GAPDH (loading control) using specific antibodies; (**B**) SERCA2a/GAPDH; (**C**) p-PLB/GAPDH; (**D**) calcineurin A/GAPDH; (**E**) p-NFATc3/GAPDH; (**F**) NFATc1/GAPDH. Mean ± SEM, n = 6 to 7 mice per group. ^*^p < 0.05 vs. WT group, ^†^p < 0.05 vs. WT-STZ group.

To explore the potential mechanisms involved in the cardiac mechanical responses to genetically knocking out cathepsin K, calcium regulatory proteins SERCA2a and the phosphorylation of PLB, as well as calcineurin/NFATs signaling were examined in whole cell lysates by Western blot (Fig. 5). Levels of SERCA2a and p-PLB were significantly reduced in diabetic WT mice. STZ challenge enhanced the calcineurin expression while decreased the NFATc3 phosphorylation without any alterations in NFATc1. These changes were reconciled in cathepsin K knockout mice.

### Metabolic signaling, apoptosis and fibrosis

**Figure 6:**
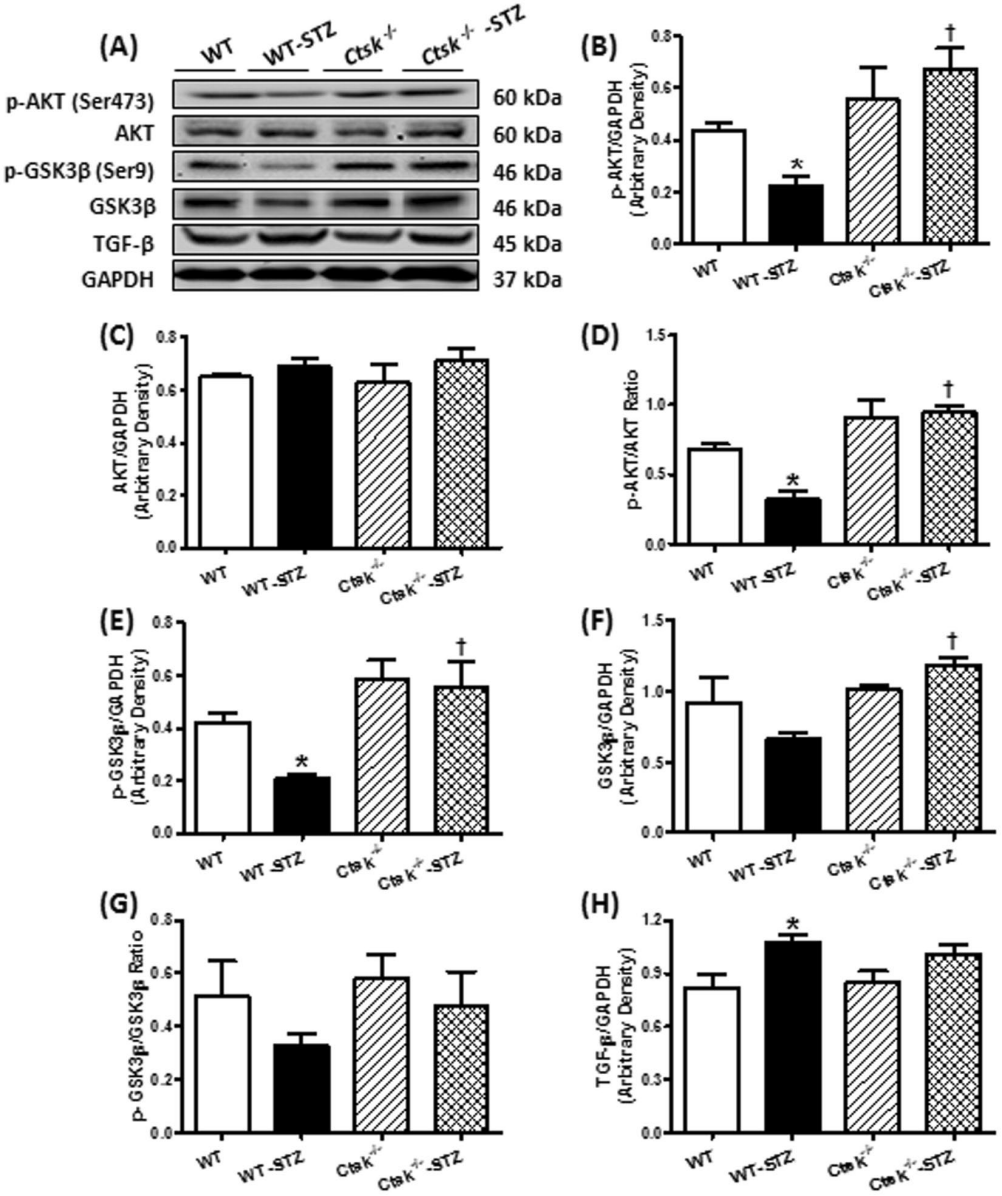
Phosphorylation of AKT, Phosphorylation of GSK3β, total AKT, total GSK3β and TGF-β in myocardium from WT and *Ctsk* ^−/−^ mice treated with or without streptozotocin. (**A**) Representative gel blots of p-AKT, AKT, p-GSK3β, GSK3β, TGF-β and GAPDH (loading control) using specific antibodies; (**B**) p-AKT/GAPDH; (**C**) AKT/GAPDH; (**D**) p-AKT/AKT Ratio; (**E**) p-GSK3β/GAPDH; (**F**) GSK3β/GAPDH; (G) p-GSK3β/GSK3β Ratio (H) TGF-β/GAPDH. Mean ± SEM, n = 6 to 7 mice per group. ^*^p < 0.05 vs. WT group, ^†^p < 0.05 vs. WT-STZ group.

**Figure 7:**
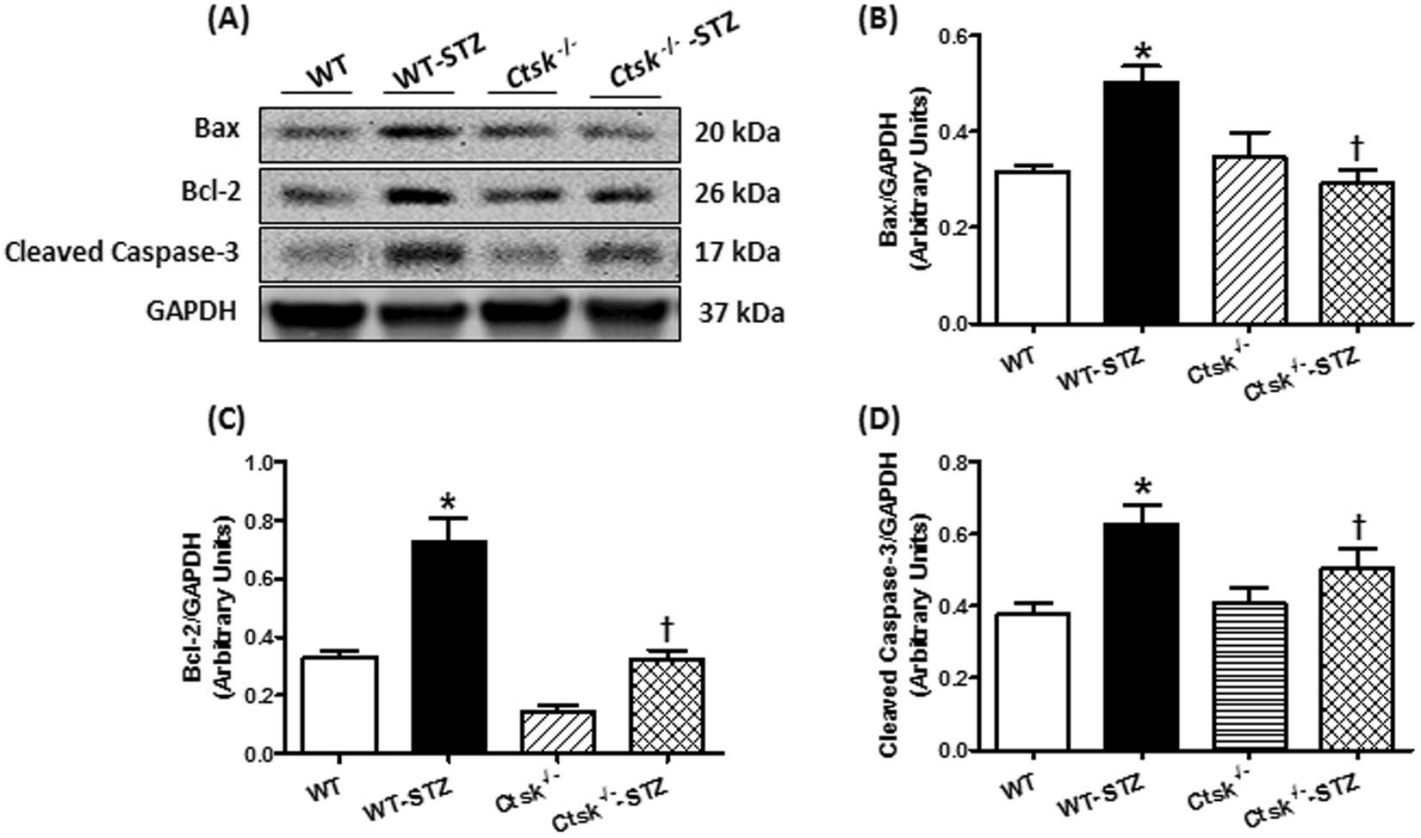
Apoptosis markers Bax, Bcl-2 and cleaved caspase-3 expression in myocardium from WT and *Ctsk* ^−/−^ mice treated with or without streptozotocin. (**A**) Representative gel blots of Bax, Bcl-2, cleaved caspase-3 and GAPDH (loading control) using specific antibodies; (**B**) Bax/GAPDH; (**C**) Bcl-2/GAPDH; (**D**) cleaved caspase-3/GAPDH; Mean ± SEM, n = 6 to 7 mice per group. ^*^p < 0.05 vs. WT group, ^†^p < 0.05 vs. WT-STZ group.

To determine the consequence of knocking out cathepsin K on metabolic signaling, apoptosis, and ECM remodeling in the myocardium, we measured protein expression and phosphorylation of AKT and GSK3β, apoptotic markers Bax, Bcl-2 and cleaved caspase-3, as well as TGF-β. Western blot analysis showed that diabetes dampened phosphorylation of AKT and GSK3β, p-AKT-to-AKT ratio and p-GSK3β-to-GSK3β ratio, and enhanced expression of Bax, Bcl-2, cleaved caspase-3 and TGF-β, which was mitigated in the cathepsin K knockout. There was no change in protein expression of AKT or GSK3β for either diabetes or cathepsin K knockout conditions (Figs 6 and 7).

### Effects of pharmacological inhibitors on glucose-induced apoptosis and oxidative stress in H9c2 cells

**Figure 8:**
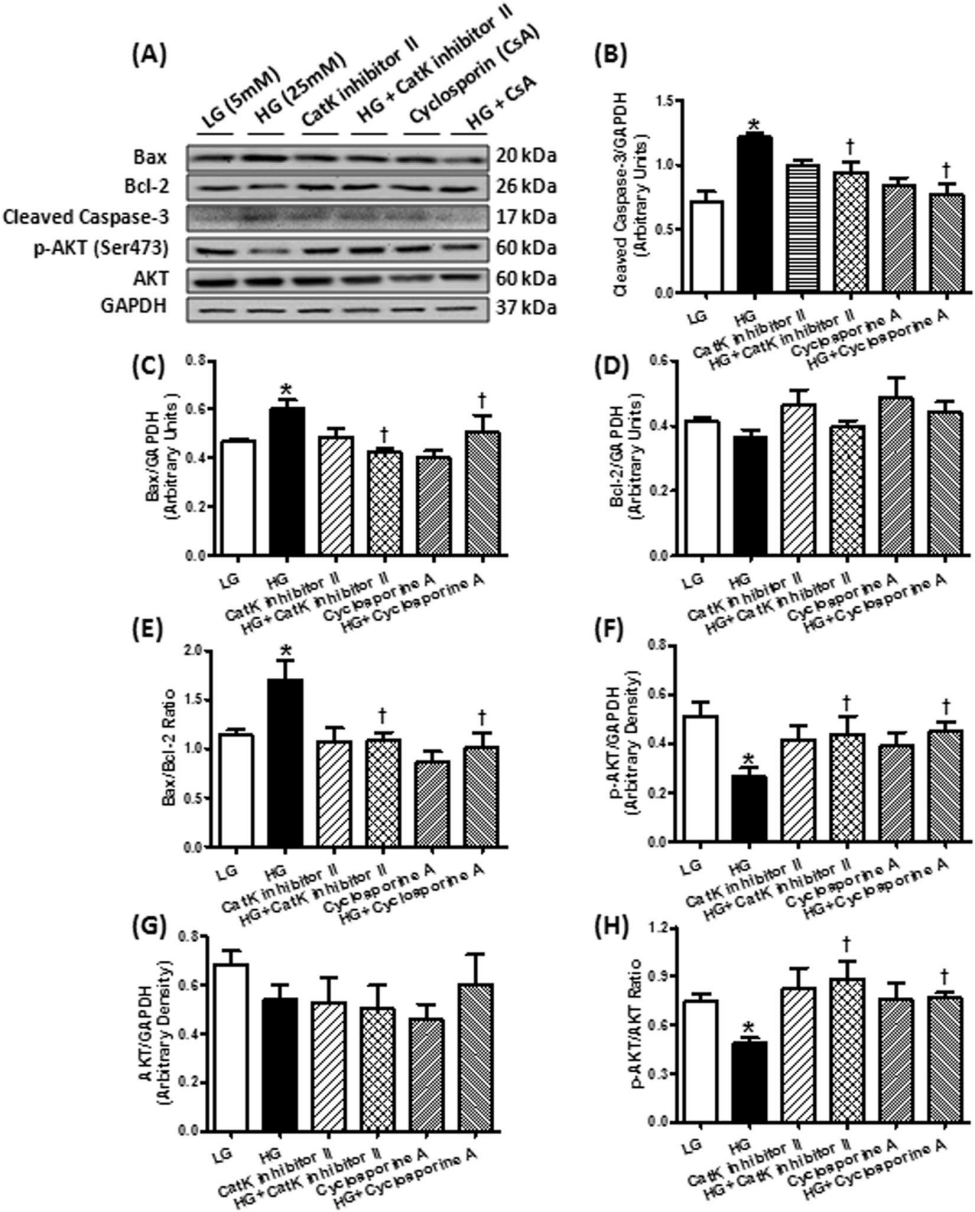
Western blot analysis of Bax, Bcl-2, cleaved caspase-3, phosphorylation of AKT, AKT and GAPDH (loading control) in H9c2 cells treated with 25 mM high glucose in the present or absent of cathepsin K inhibitor CatK I-II or calcineurin inhibitor CsA. (**A**) Representative gel blots of Bax, Bcl-2, cleaved caspase-3, p-AKT, ATK and GAPDH (loading control) using specific antibodies; (**B**) cleaved caspase-3/GAPDH; (**C**) Bax/GAPDH; (**D**) Bcl-2/GAPDH; (**E**) Bax/Bcl-2 ratio; (**F**) p-AKT/GAPDH; (**G**) AKT/GAPDH; (**H**) p-AKT/AKT ratio. ^*^p < 0.05 vs. LG group, ^†^p < 0.05 vs. HG group.

**Figure 9:**
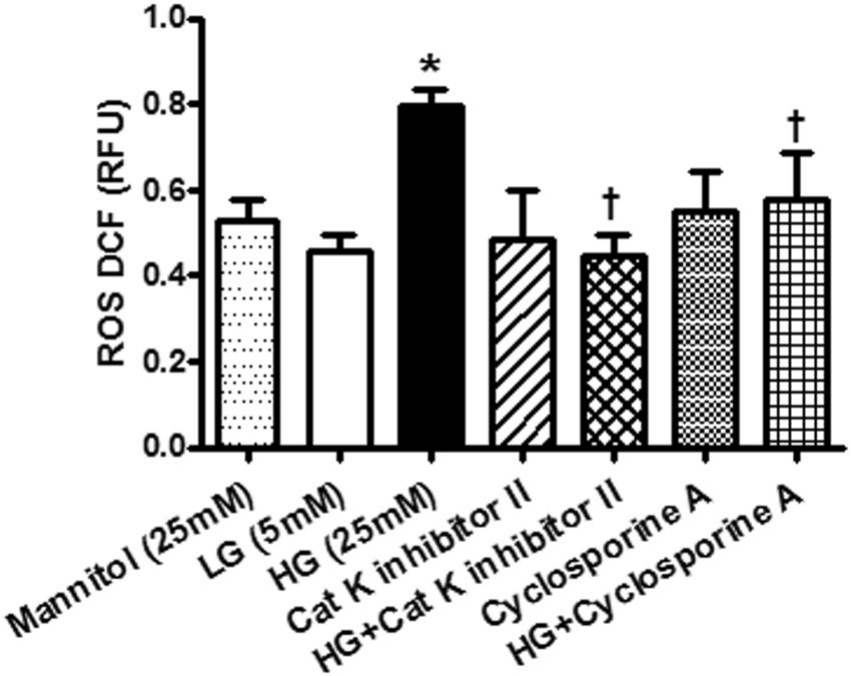
Reactive oxygen species (ROS) levels in H9c2 cells treated with 25 mM high glucose in the present or absent of cathepsin K inhibitor CatK I-II or calcineurin inhibitor CsA. ^*^p < 0.05 vs. LG group, ^†^p < 0.05 vs. HG group.

To further examine the role of cathepsin K in apoptosis and oxidative stress, cultured H9c2 cells were subjected to high-glucose (25 mM) condition in the absence or presence of a cathepsin K inhibitor II (Cat K I-II). Protein levels for AKT, phosphorylation of AKT, apoptotic markers, as well as reactive oxygen species (ROS) were evaluated. Furthermore, we evaluated the contribution of NFAT pathway, which included a pharmacological inhibitor of calcineurin cyclosporine A (CsA) in these studies. Western blot results in Fig. 8 and ROS determination in Fig. 9 shows that treatment with high-glucose upregulated cleaved caspase-3, Bax, Bax/Bcl-2 ratio, ROS level, decreased p-AKT without influencing pan AKT and expression of Bcl-2. Inhibiting cathepsin K activity or calcineurin using their respective pharmacological inhibitors Cat K I-II or CsA markedly attenuated these effects.

## Discussion

Dilated cardiomyopathy is a cardiovascular complication in diabetic subjects, which eventually leads to heart failure^1, 2^. In addition, diabetes is associated with structural and functional changes within the heart. These adaptations result in diastolic and systolic dysfunction and oxidative damage ending in a decompensated state. Despite the availability of several therapeutic options to treat diabetes and cardiac dysfunction, the prevalence of diabetic cardiomyopathy is on the rise. Therefore, identifying and characterizing pharmacological agents to treat or alleviated diabetes-associated cardiac dysfunction therefore represents an important clinical need. To our knowledge, this is the first study to show that knocking out cathepsin K confers protection against diabetes-associated cardiac complications. Specifically, we showed that knocking out cathepsin K alleviates diabetes-induced hyperglycemia, alterations in energy metabolism, and “normalizes” cardiac structure and functional anomalies. Furthermore, cathepsin K deficiency suppressed diabetes-associated myocardial apoptosis, oxidative stress and calcineurin/NFATs signaling. In cultured cardiomyocytes, glucose toxicity perturbed intracellular Ca^2+^ homeostasis due to alterations of calcium-related proteins. These changes were reconciled by deletion of cathepsin K. At the molecular level, cathepsin K activated calcineurin, facilitating the nuclear translocation of transcription factors NFAT and subsequent apoptosis.

We had previously shown that genetic knockout of cathepsin K protects against high-fat diet and abdominal-aortic constriction-induced cardiac hypertrophy and dysfunction^9, 10^. However, in both these models, it is uncertain whether cathepsin K knockout has a direct effect on cardiac anomalies associated with overt hyperglycemia as the former is a model for insulin resistance whereas the latter is a model for pressure-overload. In this study, we treated the mice with streptozotocin, which results in the destruction of the pancreatic beta cells leading to type I diabetes. Streptozotocin-induced diabetic mice typically exhibit dilated cardiomyopathy, unlike the high-fat diet and abdominal aortic constriction models used previously, which results in hypertrophic cardiomyopathy. Previous studies have shown that inhibition of cathepsin K attenuates obesity-induced increase in serum glucose and insulin, and improves systemic glucose utilization^6, 11, 12^, which is confirmed by our current study wherein cathepsin K knockout mice had lower serum glucose levels compared to the wildtype mice following STZ injection. Metabolic analysis revealed that the volume of O_2_ consumption, volume of CO_2_ production, the total activity and heat generation were significantly diminished in STZ-challenged mice, and these effects were dramatically attenuated in cathepsin K knockout mice. Thus indicating that cathepsin K deletion may contribute to the augmentation of metabolism, which has been suggested in studies from the group of Funicello and coworkers^11^. Additionally, STZ-injection significantly reduced the body weight gain, and enlarged cardiomyocyte cross-sectional area, which were mitigated by cathepsin K knockout. Consistent with previous observations, in our studies, STZ-treatment resulted in enlarged ventricular chambers^13, 14^. Genetic deletion of cathepsin K attenuated diabetes-associated changes in cardiac posterior wall thickness, increase in LVEDD and LVESD, as well as the reduction in fractional shortening and heart rate. This suggests there may be a protective role of knocking out cathepsin K activity on diabetes-induced cardiac changes. In addition, our data suggests cathepsin K has a critical role in cardiac pathology, as diabetic mice lacking cathepsin K did not develop abnormalities in cardiomyocyte contractility, intracellular Ca^2+^ homeostasis, and dysregulated calcium-related proteins such as SERCA2a and PLB. While glucose toxicity elevated the levels of cathepsin K and calcineurin A and the phosphorylation of its downstream effector NFATc3, which were attenuated in the cathepsin K knockout mice. The contribution of reductions in blood glucose (by cathepsin K knockout) to the beneficial cardiac profile in the diabetic mice cannot be ruled out. A limitation of the present study is that we did not have a conditional knockout to delineate the difference. Also, previous studies have shown that streptozotocin can have direct cardiotoxic effects when administered systemically, another limitation of our study is that we cannot fully rule out the contribution of cathepsin K knockout in countering this direct effect^15, 16^.

Cardiac injury often triggers a fibrotic response which may be attributed to the activation of proteases found in cardiomyocytes, cardiac fibroblasts or enmeshed in the extracellular matrix^17^. Interstitial fibrosis can lead to both structural and functional alterations of the heart. Therefore, proteases and antiproteases have been the target of extensive investigation in a bid to understand the progression of cardiac fibrosis and hence cardiovascular disease^18, 19^. Among the proteases studied for their role in cardiac fibrosis are the matrix metalloproteinases (MMP2 and MMP9), plasminogen activator inhibitor-1, calpains, caspases and plasmin and their endogenous inhibitors^20^. In addition, cathepsin D has been shown to be play detrimental role in diabetic cardiomyopathy whereas cathepsin L has a protective effect, underscoring the unique role of each protease^21^. Our current study adds cathepsin K to the repertoire of proteases that play a critical role in regulating cardiac function, thus broadening the potential target for treating cardiovascular disease.

Calcineurin is a serine/threonine-protein phosphatase that plays a pivotal role in eliciting hypertrophic responses by activating NFAT transcription factors and contributes to the development of dilated cardiomyopathy and heart failure^22^. Calcineurin has two subunits, a catalytic subunit, calcineurin A, and a regulatory subunit calcineurin B with four intrinsic Ca^2+^-binding sites. Calcineurin A has a catalytic domain and three regulatory domains including a calmodulin (CaM) binding domain, a calcineurin B binding domain and an autoinhibitory domain^23^. Conventionally, calcineurin is activated by increased intracellular Ca^2+^ concentration, which in turn triggers a Ca^2+^/CaM complex associated with calcineurin A through CaM binding domain and calcineurin B binding domain to expose the substrate binding cleft by releasing the autoinhibitory domain^24^. Our results showed higher levels of intracellular Ca^2+^ and calcineurin A together with an upregulation of cathepsin K in diabetic heart, which could potentially trigger CaM binding to its effector domain to increase the activity of calcineurin A^25^.

Inhibition of calcineurin/NFATs activity protects cardiomyocytes from hypertrophy by suppressing of hypertrophic genes^26, 27^. Activation of calcineurin dephosphorylates cytosolic NFATs, which translocate to the nucleus leading to transcription of genes^28, 29^. NFATc2, NFATc3 and NFATc4 (NFAT3) have been demonstrated as downstream targets of calcineurin that trigger hypertrophic signaling, especially NFATc3^30–32^. NFATc1 (NFAT2) exhibits a critical role in ECM remodeling, development of heart valves and coronary vessels during cardiac maturation, and has been shown to be an essential regulator of cathepsin K by RANKL signaling^33–35^. Our data using cultured H9c2 cells suggest that cathepsin K regulates the calcineurin/NFATc3 cascade in the progression of cardiac remodeling. In addition, calcineurin also affects Ca^2+^ fluxes by mediating various Ca^2^-related proteins and channels in the heart, specifically, SERCA2a, RyR/Ca^2+^-release channels and the L-type Ca^2+^ channel, the alteration of which is important for initiating hypertrophic processes and cardiac remodeling^36^. Finally, Ca^2+^ disorder can also cause cardiomyocyte contractile dysfunction and contribute to the pathophysiology of heart failure^37^. In our studies, calcineurin inhibitor CsA attenuated high glucose mediated impairment of SERCA2a (data not shown), suggesting a crosstalk between calcineurin and Ca^2+^ regulation. A major shortcoming of our approach is that we have used H9c2 cells (which have nevertheless been widely used to study cardiac signaling) rather than primary cultures for these signaling studies owing to the challenges in maintaining viable primary cells for over 24 h in culture.

We also found that knocking out cathepsin K reversed high-glucose-induced upregulation of Bax and cleaved caspase-3, and prevented the alterations in Bcl-2, which is in agreement with earlier observations that cathepsin K is involved in modulating apoptotic signaling in cardiomyocytes^10^, and other cell types such as osteoclasts and lung fibroblasts^38^. However, the precise molecular mechanism by which cathepsin K induces apoptosis in the heart remains unclear. It was reported that high-levels of calcineurin activity is closely associated with ischemia and caspase-3-mediated apoptosis, which can be reduced by pre-incubation with calcineurin inhibitors FK506 or cyclosporine A^39^. Furthermore, overexpression of Bcl-2 suppresses activation of calcineurin/NFAT–mediated apoptosis^40^. Additionally, constitutive overexpression of truncated calcineurin A, which lacks an autoinhibitory domain, is capable to induce apoptosis in different cell types^41^.

Alteration of intracellular Ca^2+^ is an important contributor to cellular apoptosis^42^. Our *in vivo* and *in vitro* studies demonstrated that knocking out cathepsin K significantly attenuated STZ-enhanced calcineurin/NFAT signaling and reduced GSH/GSSG. Pharmacological inhibition of cathepsin K and calcineurin decreased high glucose-induced ROS generation, apoptosis, and alterations in phospho-AKT. These observations favor the notion that the targeting cathepsin K activity lowers glucose-induced oxidative stress and apoptosis which may be mediated by the remission of intracellular Ca^2+^ disturbance and restored calcineurin-induced AKT dephosphorylation, thus triggering a pro-survival mechanism. Despite the documented role of cathepsin K as a protease, we did not observe distinctive changes in cardiac fibrosis, collagen content and TGF-β in cathepsin K knockout mice. Therefore, our data suggests a potentially nontraditional role of this protease in mediating its beneficial effects. The increased activation of GSK3β through its dephosphorylation is consistent with the studies, which showed that AKT inhibits GSK3β^43^ and calcineurin dephosphorylates GSK3β at Ser9^44^. The activated GSK3β further suppresses the glycogen synthesis from glucose^45^.

In summary, these data suggest that knocking out cathepsin K deficiency mitigates diabetes-induced cardiac anomalies due to attenuation in calcineurin/NFAT signaling and subsequent reduction in cardiac oxidative stress and apoptosis. Thus, targeting cathepsin K may represent a novel strategy to treat or prevent diabetes-associated cardiac complications.

## Methods

### Animals

All studies were performed in accordance to the relevant guidelines and was approved by the University of Wyoming’s Institutional Animal Care and Use Committee. Three-month-old male cathepsin K knockout mice (*Ctsk* ^−/−^) and C57BL/6 mice (wildtype) were subjected to intraperitoneal injections of streptozotocin (STZ, 100 mg/kg/day) dissolved in 0.1 M sterile citrate buffer or vehicle for 2 consecutive days. Following 4-weeks, fasting blood glucose levels were monitored using a glucometer. Mice with fasting blood glucose levels > 13 mM were deemed diabetic^46, 47^.

### Echocardiographic analysis

Cardiac geometry and function were evaluated in anesthetized mice using a two-dimensional guided M-mode echocardiography (Sonos 5500; Phillips Medical System, Andover, MA) equipped with a 15-6 MHz linear transducer as reported previously^48^. Heart rate, diastolic wall thickness, end-diastolic dimension (EDD), and end-systolic dimension (ESD) were measured. All measurements were done from leading edge to a leading edge in accordance with the Guidelines of the American Society of Echocardiography^49^. Heart rates were averaged over 10 cardiac cycles. The percentage of LV fractional shortening and LV mass (g) were calculated as [(EDD − ESD)/EDD] × 100 and 0.8(1.04[([LVEDD + IVSd + PWd]^3^ − LVEDD^3^)]) + 0.6 respectively. Normalized LV mass was calculated as LV mass (mg)/body weight (g)^50, 51^.

### Metabolic measurement

Metabolic measurements were made using the Comprehensive Laboratory Animal Monitoring System (CLAMS™, Columbus Instruments, Columbus, OH)^52^. Four weeks after STZ-injection, WT and *Ctsk* ^−/−^ mice were individually placed in the CLAMS metabolic cages with 100 ml water and ad libitum access to food. Following acclimation, metabolic parameters including volume of carbon dioxide produced (VCO_2_), the volume of oxygen consumed (VO_2_), the respiratory exchange ratio (RER = VCO_2_/VO_2_), the caloric (heat) value and physical activity (all horizontal beam breaks in count) were determined. All the parameters were measured every 15 min for 10 hours during daytime and 10 hours during nighttime. Results that were recorded in the first and last 2-hours were not used.

### Isolation of murine cardiomyocytes

Murine cardiomyocytes were isolated as described previously^53^. After ketamine/xylazine sedation, hearts were removed and perfused with Ca^2+^-free HEPES-buffered Tyrode’s solution containing (in mM): NaCl 120, KCl 15, KH_2_PO_4_ 0.6, Na_2_HPO_4_ 0.6, NaHCO_3_ 4.6, MgSO_4_ 1.2, HEPES 10, taurine 30, glucose 10, butanedione monoxime 10 at pH 7.4, and gassed with 95% oxygen and 5% carbon dioxide. Hearts were digested with 1 mg Liberase TH (Roche Diagnostics, Indianapolis, IN, USA) in 20 ml perfusion buffer for 10–15 min to harvest the cells. Only rod-shaped myocytes with clear edges were selected for mechanical study.

### Cell shortening/relengthening

Mechanical properties of cardiomyocytes were evaluated using a SoftEdge Myocam^®^ system (IonOptix Corporation, Milton, MA). Cardiomyocytes were placed in a chamber mounted on the stage of an inverted microscope (Olympus IX-70) and superfused with a HEPES buffer containing 1 mM CaCl_2_. Myocytes were field stimulated with suprathreshold voltage at a frequency 0.5 Hz using a pair of platinum wires placed on opposite sides of the chamber connected to a FHC stimulator (Brunswick, NE). IonOptix SoftEdge software was utilized to capture cell shortening and relengthening changes. Cell shortening and relengthening were assessed using the following indices: resting cell length, peak shortening (PS), time-to-PS (TPS), time-to-90% relengthening (TR90), and maximal velocity of shortening/relengthening (±dL/dt)^54^.

### Intracellular Ca^2+^ transient

Cardiomyocytes were loaded with fura-2/AM (0.5 μM) for 15 min, and fluorescence intensity was recorded with a dual-excitation fluorescence photomultiplier system (IonOptix). Cardiomyocytes were placed onto an Olympus IX-70 inverted microscope and were imaged through a Fluor × 40 oil objective. Cells were exposed to light excited by a 75 W lamp and passed through either a 360 or a 380 nm filter, while being stimulated to contract at a frequency of 0.5 Hz. Fluorescence emissions were detected between 480–520 nm and qualitative change in fura-2 fluorescence intensity (FFI) was inferred from the FFI ratio at the two wavelengths (360/380 nm). Fluorescence decay time (single exponential) was calculated as an indicator of intracellular Ca^2+^ clearance^54^.

### Myocardial histology

Following anesthesia, hearts were excised and immediately placed in 10% neutral-buffered formalin at room temperature for 24 h. White adipose tissues were collected from both sides of their lower abdomen and the weight was determined. Following sectioning, heart tissues were dehydrated through serial alcohols and cleared in xylenes. The specimen was embedded in the paraffin and cut into 5μm sections. Slides were deparaffinized, washed once in PBS, and stained with 0.1 mg/ml Lectin-FITC conjugate (SIGMA-Aldrich, L-4895) for 2 h at room temperature in the dark^55^. Slides were then washed with PBS and mounted with Fluoromount-G mounting media (Southern Biotech, Inc., Birmingham, AL). Cardiomyocyte cross-sectional area was measured and quantified from 90 random cardiomyocytes by using a digital microscope (×400) and the Image J (version1.39 u) software. The Masson’s trichrome staining was used to detect fibrosis in heart sections using a Masson’s Trichrome stain kit^56^.

### Determination of reduced and oxidized glutathione (GSH and GSSG)

Tissue samples (~50 mg) were sonicated in picric acid and centrifuged at 13,500 × g for 20 min. One aliquot of the supernatant was used to directly measure total GSH assay and the other for GSSG. 100 μl of supernatant fractions were treated with 2 μl vinyl pyridine to scavenge GSH for the GSSG determination. The GSSG was then subtracted from the total glutathione to evaluate the GSH levels. GSH was determined by the DTNB-glutathione reductase recycling mechanism^53^.

### Collagen ELISA assay

Cardiac collagen type I level was measured using a mouse Type I Collagen Detection Kit (Chondrex, Inc., Redmond, WA) per manufacturer’s specifications.

### Detection of Reactive Oxygen Species (ROS)

Intracellular ROS levels were evaluated by measuring the changes in fluorescence intensity resulting from intracellular probe oxidation, using a membrane-permeable probe 5-(6)-chloromethyl-2′,7′-dichlorodihydrofluorescein diacetate (CM-H_2_DCFDA) (Molecular Probes, Eugene, OR, U.S.A.)^57^. H9c2 cells were grown in a clear bottom and black side 96-well plate in the Dulbecco’s Modified Eagle Medium (DMEM) and incubated for 72 h with media containing high glucose (25 mM) with or without cathepsin K inhibitor-II (Cat K I-II, 1 µM) or calcineurin inhibitor cyclosporine A (CsA, 50 nM), and loaded with 10 µM CM-H_2_DCFDA in the culture medium for 30 min at 37 °C in the dark. DCF fluorescence was measured using fluorescent plate reader (Microplate fluorometer, Spectra GEMINI-XS) with excitation wavelength at 485 nm and emission wavelength at 530 nm.

### Western blot analysis

Protein samples were prepared as described previously and separated on SDS-polyacrylamide gels, transferred electrophoretically to nitrocellulose membranes and blotted against cathepsin K (1:500), anti-phospho-phospholamban (p-PLB, 1:1,000, Ser16/Thr17), anti-SERCA2a (1:1,000), anti-calcineurin A (1:1,000), anti-NFATc1 (1:250), anti-phospho-NFATc3 (1:250), anti-glycogen synthase kinase 3 beta (GSK3β, 1:1,000), anti-phospho-GSK3β (1:1,000), anti-AKT (1:1,000), anti-phospho-AKT (1:1,000, Ser473), anti-transforming growth factor-β (TGF-β, 1:1,000), anti-cleaved caspase-3 (1:500), anti-Bcl-2 (1:1000), anti-Bax (1:1,000), anti-GAPDH (loading controls, 1:1000) and anti-α-tubulin (loading controls, 1:1000) antibodies. Blots were incubated with horseradish peroxidase (HRP)-conjugated secondary antibody (1:3000). Antigens were detected by the luminescence method. Band densities were determined using Quantity One software (Bio-Rad, version 4.4.0, ChemiDoc XRS)^48^.

### Statistical analysis

Data are presented as mean ± SEM. Statistical significance (p < 0.05) for each variable was estimated by a one-way analysis of variance (ANOVA) followed by a Turkey’s post-hoc analysis.

### Data availability

The datasets generated during and/or analyzed during the current study are available from the corresponding author upon reasonable request.

## Electronic supplementary material

Supplementary Figure 1.
