# Supplementary material for: Cathepsin K knockout protects against cardiac dysfunction in diabetic mice
Source: Sci Rep. 2017 Aug 18;7:8703. doi: 10.1038/s41598-017-09037-z (PMC5562704; doi:10.1038/s41598-017-09037-z)
Supplement: Supplementary file 1 — Supplementary Figure 1. [file 41598_2017_9037_MOESM1_ESM.doc]

**Cathepsin K knockout protects against cardiac dysfunction in diabetic mice**

Guo R, Hua Y, Rogers O, Brown TE, Ren J, Nair S.

**Supplementary Fig 1.** Histological analyses and collagen Type I measurement in hearts from WT and *Ctsk*^-/-^ mice treated with or without streptozotocin. (A-D) Representative images of Masson trichrome staining for fibrosis (× 400; scale bar = 20μm); (E-H) Representative fluorescein isothiocyanate (FITC)-conjugated wheat germ lectin staining depicting cardiomyocyte size (× 400; scale bar = 20μm); (I) Collagen type I content; (J) Quantitative cardiomyocyte cross-sectional (transverse) area from 70 cells from three mice per group. Mean ± SEM, ^*^p<0.05 vs. WT group, ^†^p<0.05 vs. WT-STZ group.
